# Editorial: Entropy in Landscape Ecology

**DOI:** 10.3390/e20050314

**Published:** 2018-04-25

**Authors:** Samuel A. Cushman

**Affiliations:** Rocky Mountain Research Station, USDA Forest Service, 2500 S Pine Knoll Dr., Flagstaff, AZ 86004, USA; scushman@fs.fed.us

**Keywords:** landscape, entropy, thermodynamics, second law, configuration, pattern

## Abstract

Entropy and the second law of thermodynamics are the central organizing principles of nature, but the ideas and implications of the second law are poorly developed in landscape ecology. The purpose of this Special Issue “Entropy in Landscape Ecology” in *Entropy* is to bring together current research on applications of thermodynamics in landscape ecology, to consolidate current knowledge and identify key areas for future research. The special issue contains six articles, which cover a broad range of topics including relationships between entropy and evolution, connections between fractal geometry and entropy, new approaches to calculate configurational entropy of landscapes, example analyses of computing entropy of landscapes, and using entropy in the context of optimal landscape planning. Collectively these papers provide a broad range of contributions to the nascent field of ecological thermodynamics. Formalizing the connections between entropy and ecology are in a very early stage, and that this special issue contains papers that address several centrally important ideas, and provides seminal work that will be a foundation for the future development of ecological and evolutionary thermodynamics.

Entropy and the second law of thermodynamics are the central organizing principles of nature, but the ideas and implications of the second law are poorly developed in landscape ecology. This seems strange given that landscape ecology explicitly focuses on understanding pattern-process relationships across scales in space and time. Every interaction between entities leads to irreversible change, which increases the entropy and decreases the free energy of the universe. Descriptions of landscape patterns, processes of landscape change, and propagation of pattern-process relationships across space and through time are all governed, constrained, and, in large part, directed by thermodynamics. 

In a recent paper about the need to integrate thermodynamics and ecological research [1] I made the following statements: (1) Improved measures of configurational entropy are needed to quantify landscape dynamics and the interactions of patterns and processes across scales of space and time. (2) It is essential to evaluate the spatio-temporal dynamics of entropy in ecological time-series. (3) Landscape ecologists should more formally associate landscape dynamics with changes in entropy and quantify the function of ecological dissipative structures. (4) A landscape maintains a dynamic equilibrium under a disturbance-succession regime through the collective emergent property of many organismal dissipative structures in interaction with abiotic drivers, such as solar energy, temperature, and moisture. (5) Linking the scale dependence of landscape dynamics to thermodynamic constraints across different ecosystem types would be central to generalizing the application of entropy in landscape ecology. (6) The linkage of the entropy principle with the concepts of resistance, resilience and recovery is critical to provide a rigorous science of sustainability. (7) The flow of energy and resulting patterns of order and disorder may result in increase or decrease in system predictability over time depending on whether the energy flow results in net decrease in entropy of the landscape or a net increase. (8) Fractal dimension is directly related to entropy, given that it is a measure of a pattern-process scaling law and the thermodynamic behavior of dissipative structures drives their emergence. (9) Scale dependence is a product of the action of dissipative structures organized across a range of scales or hierarchical levels. (10) When ecological systems are properly viewed as multiscale and hierarchically organized dissipative structures then it is clear that thermodynamic irreversibility applies to them. (11) The application of thermodynamic entropy concepts in landscape ecology has not addressed the true thermodynamic nature of the actions of dissipative structures across scales, and this has been limited by failure to measure energy transformations, changes in free energy, changes in configurational entropy of landscape mosaics. 

The purpose of this Special Issue in *Entropy* is to bring together current research on applications of thermodynamics in landscape ecology, to consolidate current knowledge and identify key areas for future research. The issue contains six articles, not including this editorial. 

First, the theme of linkage between evolution and entropy is developed in “Entropy in the tangled nature model of evolution. [2]” Roach et al. provide a conceptual model of the role of entropy in evolving ecological systems based on the Tangled Nature Model, in which they derived two measures of configurational entropy and show that both measures applied to the system increase over time, while that of simulated biological organisms decrease. This simulation provides a nice illustration of the process by which dissipative structures can build organization at the expense of the wider system through simple random processes, as argued in the Cushman paper described above. They use this conceptual model to discuss the relationship between entropy and the niche functional space of ecosystems, as well taxonomic configurations that emerge from different niche partitioning and functionality. 

In “Horton ratios link self-similarity with maximum entropy of eco-geomorphological properties in stream networks,” Milne and Gupta provide the first formal linkage between the concept of fractal self-similarity and entropy [3], that was mentioned by Cushman [1]. Specifically, they use the example of branching stream networks to show the linkage between Budyko theory and Horton scaling. They show that there is an optimum entropy that is consistent with the highest biodiversity, and show that Horton ratios are equivalent to Lagrange multipliers. This highly novel linkage between entropy and fractal geometry is groundbreaking in a number of respects. The authors note that “the entropy-Horton framework informs questions of biodiversity, resilience to perturbations in water supply, changes in potential evapotranspiration, and land use changes that move ecosystems away from optimal entropy with concomitant loss of productivity and biodiversity.” This formal linkage between fractal complexity and entropy I believe is immensely important, and provides a foundation for further development of the linkage between complexity theory and entropy, and I am very excited to see this line of research continue to illuminate how undirected irreversible thermodynamic change leads to emergence of all ecological complexity.

Cushman et al. [4] proposed the first entropy-based measure of landscape structure, and argued that refocusing landscape pattern analysis in the context of thermodynamic processes would be important to find fundamental theories in the emergence of ecological patterns and connecting them with fundamental physical drivers. Two papers extend this line of work. First, in “Calculation of configurational entropy in complex landscapes [5],” I extend the original methods of calculating the entropy of a landscape lattice to address a number of practical limitations. In [4] I showed that the entropy of a landscape mosaic can be calculated using the Boltzmann equation, with the entropy of a lattice mosaic equal to the logarithm of the number of ways a lattice with a given dimensionality and number of classes can be arranged to produce the same total amount of edge between cells of different classes. However, that work seemed to also suggest that the feasibility of applying this method to real landscapes was limited due to intractably large numbers of possible arrangements of raster cells in large landscapes. In this paper I extend that work by showing that: (1) the proportion of arrangements rather than the number with a given amount of edge length provides a means to calculate unbiased relative configurational entropy, obviating the need to compute all possible configurations of a landscape lattice; (2) the edge lengths of randomized landscape mosaics are normally distributed, following the central limit theorem; and (3) given this normal distribution it is possible to fit parametric probability density functions to estimate the expected proportion of randomized configurations that have any given edge length, enabling the calculation of configurational entropy on any landscape regardless of size or number of classes. I evaluate the boundary limits (4) for this normal approximation for small landscapes with a small proportion of a minority class and show it holds under all realistic landscape conditions. I further (5) demonstrate that this relationship holds for a sample of real landscapes that vary in size, patch richness, and evenness of area in each cover type, and (6) I show that the mean and standard deviation of the normally distributed edge lengths can be predicted nearly perfectly as a function of the size, patch richness and diversity of a landscape. Finally, (7) I show that the configurational entropy of a landscape is highly related to the dimensionality of the landscape, the number of cover classes, the evenness of landscape composition across classes, and landscape heterogeneity. These advances provide a means for researchers to directly estimate the frequency distribution of all possible macrostates of any observed landscape, and then directly calculate the relative configurational entropy of the observed macrostate, and to understand the ecological meaning of different amounts of configurational entropy. 

In “Entropies of the Chinese land use/cover change from 1990 to 2010 at a county level [6],” Fan et al. evaluate the use of Shannon, Tenyi and Tasllis entropies as measures of landscape complexity. They show that Shannon entropy reflects the volatility of the changes to land use/land cover, and that Renyi and Tsallis entropies also have this functions when they have positive parameter values, and that they reflect other attributes of pattern when they have negative parameter values. The authors evaluate temporal change in the time series of landscape entropy in China and observe accelerating landscape entropy and identify spatial regions with clear differences in the rate and pattern of change in landscape entropy. This, to my knowledge, is the first example of a temporal and spatial analysis of the different rates and patterns of change in landscape entropy, and as such is an important milestone in the development of entropy based landscape pattern analysis and assessment of landscape dynamics from a thermodynamic viewpoint.

In their insightful paper “Radiative entropy production along the paludification gradient in the southern taiga [7],” Kuricheva et al. propose that entropy production is a measure of ecosystem and landscape stability in a changing environment. They used calculations of the rate of radiative entropy production in three different taiga ecosystems to show that rate of radiative entropy production depends on both surface temperature and radiation absorption, but that in boreal ecosystem the radiation effect is much larger than the temperature effect. They showed that forest ecosystems have a stable rate of radiative entropy production that was not affected by seasonal variation or drought, while, in contrast, the rate of radiative entropy production in bogs is extremely sensitive to water level, and associated changes in albedo. They argue that paludification, such as associated with permafrost melting, may increase the instability of the energy balance and entropy production in the taiga landscape.

The final paper in the special issue is “Discussing landscape compositional scenarios generated with maximization of non-expected utility decision models based on weighted entropies [8],” by Casquiho and Castro Rego, which focuses on how the entropy concept can be used to develop optimal solutions of landscape composition in a landscape planning context. They propose a two-dimensional decision-space consisting of economic value and landscape diversity. The authors use utility valuations combined with weighted entropies respectively incorporating rarity factors associated to Gini-Simpson and Shannon measures to develop an optimization approach for decision making. The found that using Shannon weighted entropy and a square root utility function provided the best route to optimized lands use decisions. This paper is novel as it is among the first to attempt to formally combine measures of landscape entropy with economic utility functions to balance tradeoffs between economic benefit and environmental cost, and address the problem of externalities.

Collectively these papers provide a broad range of contributions to the nascent field of ecological thermodynamics. For example, the formal linkage of self-similarity and entropy is a hugely important theoretical advance that I think will pave the way for further integration of the ideas of entropy and complexity in ecological systems. Similarly, a number of the papers propose and illustrate new and powerful ways to measure the entropy of ecological systems, which is a fundamental task to integrating thermodynamics with ecological theory. One of the papers in the special issue addresses the relationships between evolution and entropy, which I believe is a topic ripe for development, and holds the potential to completely reframe how scientists think about organisms and ecological systems. Formalizing the connections between entropy and ecology are in a very early stage, but it is my belief that this special issue contains papers that address several centrally important ideas, and provides seminal work that will be a foundation for the future development of ecological and evolutionary thermodynamics.
